# Esophageal Injury Risk Factors in Patients With Atrial Fibrillation Undergoing Catheter Ablation

**DOI:** 10.1002/joa3.70259

**Published:** 2026-02-09

**Authors:** Yun Young Choi, Hyuk Soon Choi, Joo Hee Jeong, Chang Ok Seo, Yun Gi Kim, Jaemin Shim, Jong‐Il Choi, Young‐Hoon Kim

**Affiliations:** ^1^ Cardiovascular Center Seoul National University Bundang Hospital, Seoul National University College of Medicine Seongnam Korea; ^2^ Division of Gastroenterology and Hepatology Korea University College of Medicine, Korea University Medicine Seoul Republic of Korea; ^3^ Division of Cardiology Korea University College of Medicine and Korea University Medical Center Seoul Republic of Korea

**Keywords:** atrial fibrillation, catheter ablation, cryoballoon, esophageal injury

## Abstract

**Background and Aims:**

Esophageal injury (EI) is a potentially serious complication of catheter ablation (CA) for atrial fibrillation (AF). However, data on its incidence and risk factors, particularly after radiofrequency catheter ablation (RFCA) and cryoballoon ablation (CBA), remain limited. This study aimed to identify predictors of EI in patients undergoing RFCA and CBA.

**Methods:**

In this retrospective study, patients with AF who underwent de novo RFCA or CBA between December 2019 and April 2022 were evaluated. All underwent EGD within 2 days post‐ablation. EI severity was graded, and clinical predictors were analyzed using multivariate logistic regression.

**Results:**

Among 584 patients (mean age 62.8 ± 11.2 years, 68.6% male), 30 (5.1%) developed EI (RFCA: 5.0%, CBA: 6.3%). Most injuries were mild (Class I), and all resolved with proton pump inhibitor therapy. Compared with those without EI, affected patients had significantly lower body mass index (BMI) (24.5 ± 3.9 vs. 25.9 ± 3.4 kg/m^2^, *p* = 0.030) and reduced ejection fraction (EF) (48.9% ± 8.7% vs. 52.8% ± 6.8%, *p* = 0.023). Multivariate analysis identified BMI ≤ 24 kg/m^2^ (odds ratio [OR], 3.67; 95% confidence interval [CI], 1.69–7.96) and EF ≤ 40% (OR, 4.08; 95% CI, 1.54–11.56) as independent risk factors.

**Conclusions:**

Esophageal injury after catheter ablation for atrial fibrillation is not rare, but usually mild. Lower BMI and reduced LVEF were independent risk factors, regardless of ablation type. These results support selective endoscopic screening in high‐risk patients to enable early detection and prevent severe complications.

## Graphical abstract

Low BMI and reduced LVEF were independently associated with esophageal injury following catheter ablation for atrial fibrillation. All lesions were located near the left pulmonary vein and showed complete healing on follow‐up endoscopy.

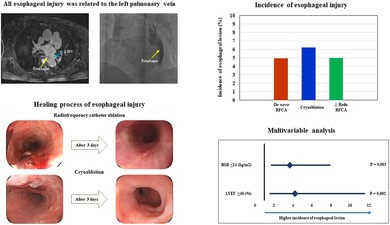

## 1 Introduction

The esophagus lies slightly left of midline, positioned between the trachea and vertebral column. It is contact with the endocardial surface of the left atrium (LA) is minimal, typically less than 5 mm in length and 15 mm in width along the posterior LA wall [1]. Atrioesophageal fistula (AEF) is a rare but life‐threatening complication of catheter ablation (CA) for atrial fibrillation (AF), occurring in up to 0.2% of patients and carrying a mortality rate as high as 80% [2]. Esophageal injuries (EI) and AEF are most frequently associated with ablation near the left inferior pulmonary vein (PV) [3, 4]. Given the thermal injury mechanism, strategies such as esophageal temperature monitoring and contact force modulation are employed to reduce the risk of EI. Notably, elevated esophageal temperature exceeding 41°C is associated with a significantly increased risk, with the likelihood of injury rising by 1.36‐fold for each 1°C increment [5]. In addition to direct thermal damage, EI progression may be influenced by secondary mechanisms, including reflux esophagitis and gastric hypomotility. Injury to large vagal nerve fibers may impair lower esophageal sphincter relaxation, exacerbating acid reflux and promoting mucosal injury. Reflux‐related inflammation may further erode esophageal tissue, potentially contributing to fistula formation [6]. This delayed pathogenesis may explain the typical 2–4 weeks latency observed in AEF development. Accordingly, early recognition and intervention are critical to mitigate progression and reduce associated mortality.

Proton pump inhibitors (PPI) are effective in managing reflux esophagitis by reducing gastric acid secretion, promoting mucosal healing, and minimizing the size of iatrogenic ulcers [7]. Early detection of EI followed by prompt administration of high‐dose PPI therapy may prevent lesion progression by controlling acid‐related inflammation. Accordingly, esophagogastroduodenoscopy (EGD) is a valuable tool for the early identification of EI after CA. In this study, we evaluated patients who underwent EGD and investigated the risk factors for EI after CA, including radiofrequency catheter ablation (RFCA) and cryoballoon ablation (CBA).

## 2 Methods

### 2.1 Study Population and Design

All consecutive patients who underwent RFCA or CBA for AF at a single tertiary center between December 2019 and April 2022 were retrospectively analyzed. Patients who underwent EGD within 2 days following the ablation procedure were included. The study protocol was approved by the Institutional Review Committee (no. 2020AN0165), and the requirement for informed consent was waived due to the retrospective nature of the analysis.

### 2.2 Electrophysiological Studies and Catheter Ablation

Prior to sedation, barium esophagography was performed to localize the esophagus. The esophageal position was visualized using chest fluoroscopy in both 35° right and 35° left anterior oblique projections following barium contrast administration. Three‐dimensional (3D) reconstruction using computed tomography (CT) was employed to accurately delineate the esophageal course for both RFCA and CBA procedures. All ablation procedures were conducted at our institution [8]. LA geometry and ablation were carried out using a 3D mapping system with NavX EnSite (Abbott, St. Paul, MN, USA). To minimize the risk of esophageal lesions, ablation near the posterior left PV was performed only after confirming the esophageal position via barium esophagography. Radiofrequency ablation was delivered at 25 W over the posterior LA wall and 30 W for a duration of 20–30 s at other sites without use of Ablation Index (AI) or Lesion Size Index (LSI). In high‐power short‐duration (HPSD) ablation, energy was delivered at 50 W for less than 7 s based on electrogram‐guided targets. For CBA, second‐generation cryoablation (Arctic Front Advanced Cardiac Cryoablation Catheter; Medtronic) was used to achieve PV isolation (PVI). Each PV was targeted with a single 28 mm cryoballoon. If the initial freeze failed to achieve isolation, additional applications were performed until isolation was confirmed. PV isolation was achieved using a circumferential antral ablation approach. Electrical isolation was confirmed by entrance and exit block. Additional ablation was performed for conduction gaps or non‐PV triggers based on physician's discretion.

### 2.3 Esophagogastroduodenoscopy

All patients underwent scheduled EGD performed by an experienced gastroenterologist on the day following CA. Procedures were conducted using a standard endoscopic system (EVIS LUCERA ELITE CV‐290; Olympus Medical Systems). Thermal injuries were documented via endoscopic imaging, which was independently reviewed by a second gastroenterologist to confirm the presence and characteristics of EI. The severity of EI was classified using the Kansas City Classification [9] ranging from erythema or submucosal blood clots (Class I) to superficial and deep ulcerations (Classes II–III), and, in rare cases, transmural perforation with or without LA communication (Class IV) (Table S1). Patients with persistent EI were treated with high‐dose proton pump inhibitor (PPI) (esomeprazole 40 mg twice daily) for at least two weeks. For those with confirmed EI, follow‐up EGD was performed 3–5 days later to assess mucosal healing. During this interval, patients were permitted to take small sips of water.

### 2.4 Statistical Analysis

Continuous variables are presented as mean ± standard deviation and were compared between groups using Student's *t*‐test. Categorical variables were analyzed using the chi‐squared test or Fisher's exact test, as appropriate. Univariate logistic regression analysis was initially performed to identify factors associated with EI, followed by multivariate logistic regression to determine independent predictors. All statistical tests were two‐tailed, and a *p*‐value < 0.05 was considered statistically significant. Statistical analyses were conducted using SPSS software (version 25.0; IBM Corp., Armonk, NY, USA).

## 3 Results

### 3.1 Study Patients

**FIGURE 1:**
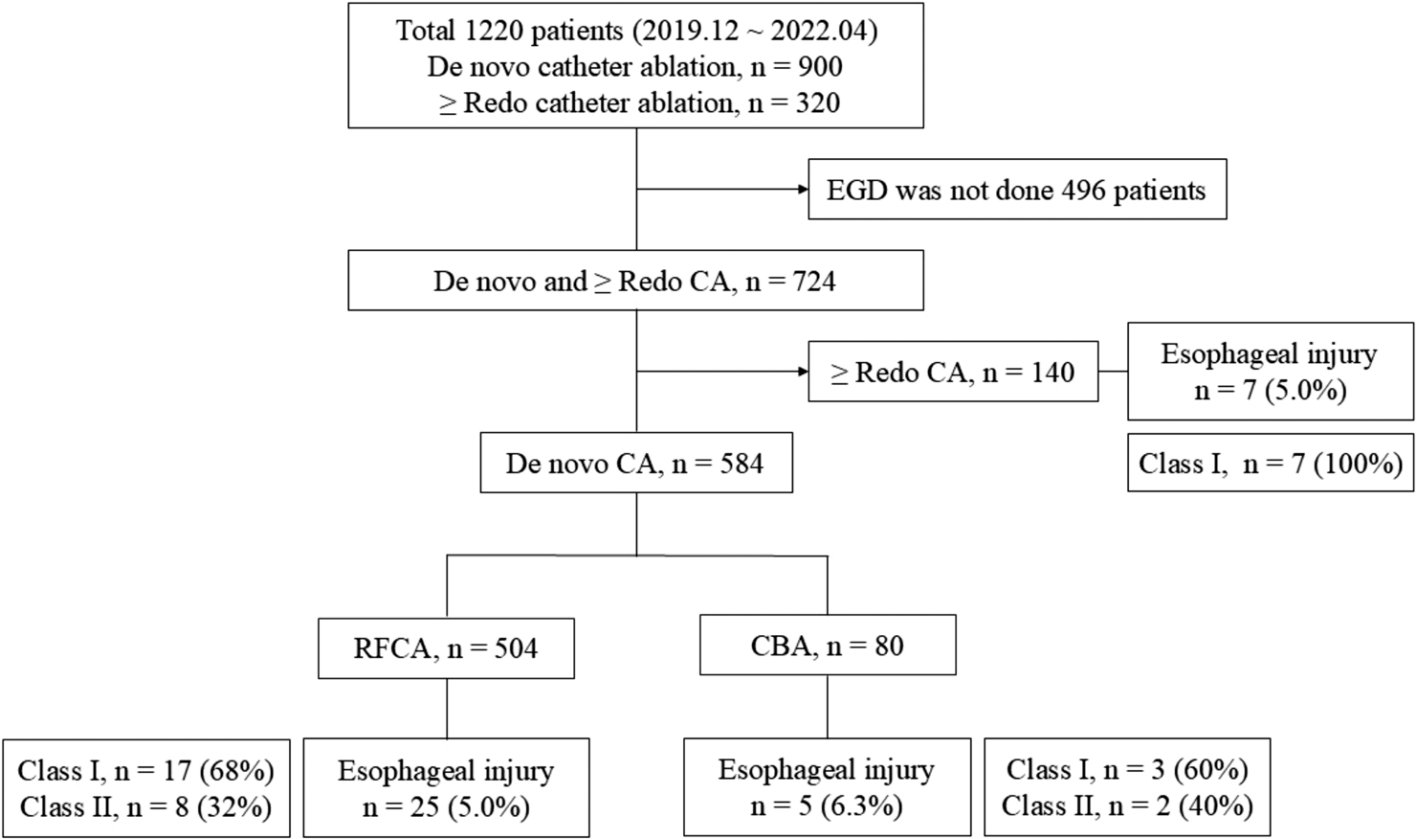
Flow chart of the patient selection process. Among 1220 consecutive patients who underwent RFCA between December 2019 and April 2022. Among those who underwent de novo CA, 504 received RFCA and 80 patients underwent CBA. A total of 30 patients (5.1%) were diagnosed with esophageal injury (EI): 25 in the RFCA group (5.0%) and 5 in the CBA group (6.3%). CA, catheter ablation; CBA, cryoballoon ablation; EGD, esophagogastroduodenoscopy; RFCA, radiofrequency catheter ablation.

Among 1220 patients who underwent RFCA between December 2019 and April 2022, 724 received post‐procedural EGD, excluding 496 patients due to limited EGD availability during the early COVID‐19 period. Of these, 584 underwent de novo CA (504 RFCA, 80 CBA). Esophageal injury (EI) was identified in 30 patients (5.1%): 25 after RFCA (5.0%) and 5 after CBA (6.3%). Most lesions were mild, with 68% in the RFCA group and 60% in the CBA group classified as Class I per the Kansas City Classification (Figure 1).

### 3.2 Baseline Characteristics of Patients With and Without Esophageal Injury

Table 1 presents the baseline characteristics of the patients with and without EI following de novo CA. The mean age of the overall cohort was 62.8 ± 11.2 years, and 68.6% were male. Patients who developed EI had significantly lower body mass index (BMI) (24.5 ± 3.9 vs. 25.9 ± 3.4 kg/m^2^, *p* = 0.030) and reduced left ventricular ejection fraction (LVEF) (48.9% ± 8.7% vs. 52.8% ± 6.8%, *p* = 0.023) compared to those without EI. Comorbidities and echocardiographic findings were not significantly different between the two groups. Preprocedural CT was available for all patients and was used to assess esophageal anatomical location. As summarized in Table S2, the esophagus was located to the left of the LA in the vast majority of cases, 488 (96.8%) of 504 RFCA patients and 79 (98.8%) of 80 CBA patients. Importantly, all patients who developed EI had a left‐sided esophagus, demonstrating a consistent anatomical pattern among affected individuals.

**TABLE 1:** Baseline characteristics of patients with or without esophageal injury.

Variables	Total	EI (−)	EI (+)	*p*
	(*n* = 584)	(*n* = 554)	(*n* = 30)	
Age, years	62.8 ± 11.2	62.8 ± 11.3	62.6 ± 8.8	0.905
Male, *n* (%)	401 (68.6)	379 (67.8)	20 (66.7)	0.902
Body weight (kg)	71.9 ± 13.4	72.1 ± 13.4	70.0 ± 14.6	0.411
Height (cm)	166.5 ± 9.8	166.4 ± 9.8	168.6 ± 9.6	0.215
BMI (kg/m^2^)	25.8 ± 3.5	25.9 ± 3.4	24.5 ± 3.9	0.030
Paroxysmal atrial fibrillation, *n* (%)	249 (42.6)	239 (43.1)	10 (33.3)	0.284
Congestive heart failure, *n* (%)	159 (27.2)	150 (27.1)	10 (30)	0.727
Hypertension, *n* (%)	338 (57.9)	320 (57.8)	18 (60)	0.809
Diabetes, *n* (%)	100 (17.1)	95 (17.1)	5 (16.7)	0.946
Previous stroke, *n* (%)	54 (9.2)	50 (9)	4 (13.3)	0.428
Vascular disease, *n* (%)	26 (4.5)	23 (4.2)	3 (10)	0.308
CHA_2_DS_2_‐VASc score	2.13 ± 1.4	2.13 ± 1.4	2.20 ± 1.6	0.779
RFCA, *n* (%)	504 (86.3)	479 (95.0)	25 (5.0)	0.628
Cryoablation, *n* (%)	80 (13.7)	75 (93.7)	5 (6.3)	0.628
Echo findings				
Left ventricular ejection fraction (%)	52.6 ± 6.9	52.8 ± 6.8	48.9 ± 8.7	0.023
Left atrium diameter (mm)	42.8 ± 6.3	42.8 ± 6.4	43.2 ± 5.2	0.774
E/e'	9.4 ± 4.7	9.5 ± 4.8	8.6 ± 2.0	0.305
Pulmonary artery pressure (mmHg)	30.7 ± 8.1	30.6 ± 8.2	32.4 ± 6.3	0.240
LVEDD (mm)	46.8 ± 5.0	46.7 ± 5.0	48.3 ± 4.6	0.100
Left atrial volume (mL)	115.7 ± 39.2	116 ± 39.7	111.5 ± 28.2	0.541
TEE before RFCA, *n* (%)	107 (18.3)	98 (17.7)	9 (30)	0.165
Ablation, *n* (%)				
CFAE	10 (1.7)	10 (1.8)	0 (0)	0.459
Roof	27 (4.6)	25 (4.5)	2 (6.7)	0.585
Anterior	46 (7.9)	46 (8.3)	0 (0)	< 0.001
Posterior wall ablation	39 (6.7)	37 (6.7)	2 (6.7)	0.998
Perimitral isthmus	24 (4.1)	23 (4.2)	1 (3.3)	0.826
SVC	68 (11.6)	66 (12)	2 (6.7)	0.384
CTI	401 (68.7)	380 (68.6)	21 (70)	0.873
Left PV additional ablation	172 (29.5)	159 (28.7)	13 (43.3)	0.130

Abbreviations: BMI, body mass index; CFAE, complex fractionated atrial electrogram; CTI, cavotricuspid isthmus; EI, esophageal injury; LVEDD, left ventricular end‐diastolic diameter; PV, pulmonary vein; RFCA, radiofrequency catheter ablation; SVC, superior vena cava; TEE, transesophageal echocardiography.

### 3.3 Comparison of Catheter Ablation Modalities: Radiofrequency and Cryoballoon

Among patients who developed EI, all individuals in the CBA group were male. Paroxysmal AF was significantly more common in the CBA group compared to the RFCA group (80% vs. 24%, *p* = 0.014). Furthermore, the LA diameter was significantly larger among patients who underwent RFCA compared to those who underwent CBA (44.1 ± 4.8 vs. 35.8 ± 4.4 mm, *p* = 0.024). In terms of comorbidities, patients who developed EI after RFCA had higher rates of diabetes mellitus, prior stroke, and congestive heart failure compared with those who developed EI following CBA (Table S3).

In patients who underwent CBA, detailed procedural parameters were analyzed by PV segment. Table S4 compares time to isolation, nadir temperature, number of applications, and freeze duration between patients with and without esophageal injury. No significant differences were observed, although lower nadir temperatures and a greater number of applications were numerically more frequent in some veins among patients with EI.

### 3.4 Type of Esophageal Injury

**FIGURE 2:**
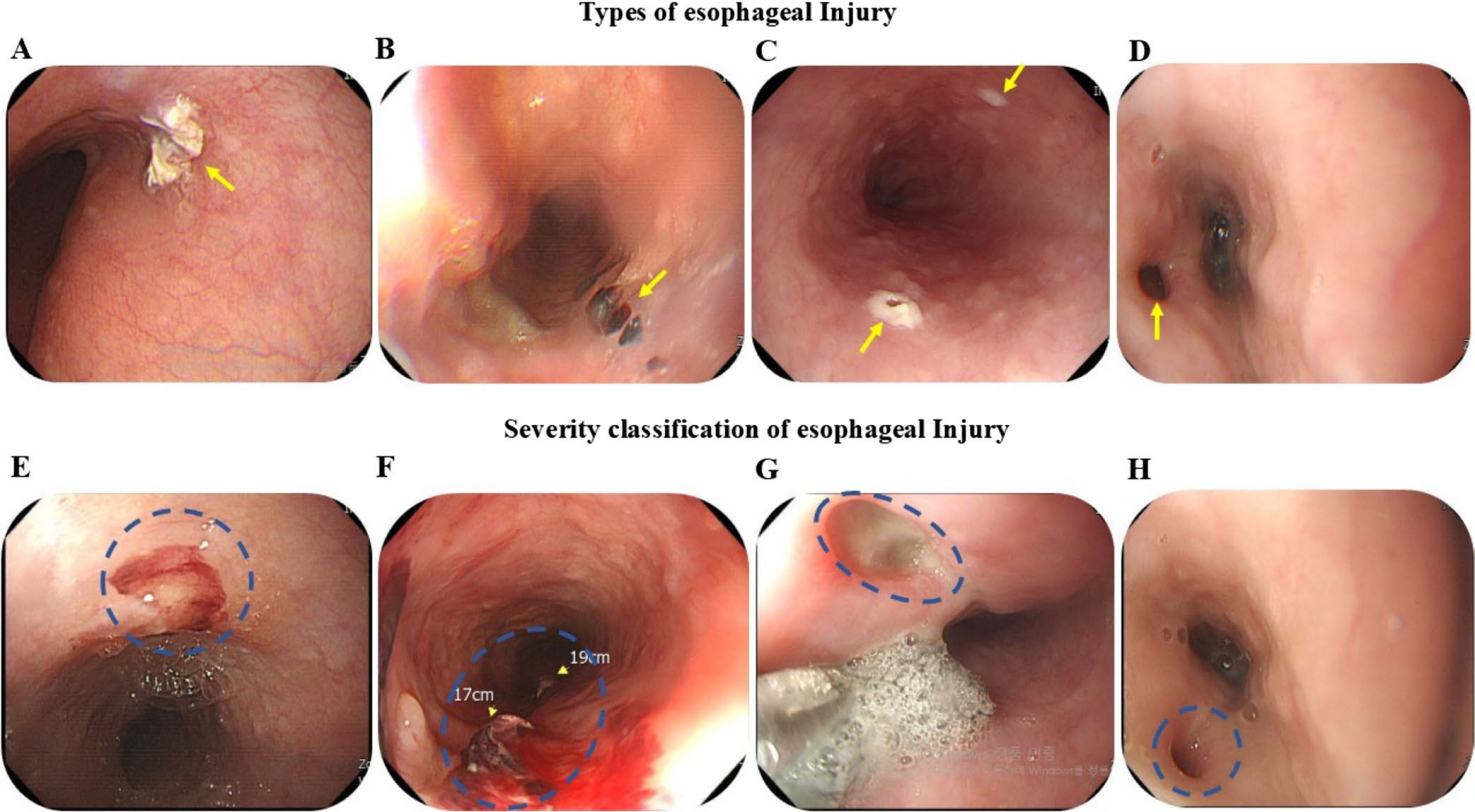
Types and severity of esophageal injuries following catheter ablation. EI typically appears as an acute lesion on the anterior esophageal wall, distant from the lower esophageal sphincter. (A–D) show representative lesion types: (A) Solitary lesion; (B) Linear lesion; (C) Kissing lesion; (D) Atrial‐esophageal fistula. Yellow arrows indicate the lesion sites. (E, F) illustrate severity grading based on the Kansas City Classification: (E) Minimal lesions such as erythema, (F) Superficial ulcer, (G) Deep ulcer, and (H) Perforation with or without fistulous communication with the atria. The blue dotted circle represents the EI. CA, catheter ablation; CBA, cryoballoon ablation; EI, esophageal lesion; HPSD, high‐power short‐duration; RFCA, radiofrequency catheter ablation.

Figure 2 illustrates the morphologies and severity grades of EI following CA. EI was primarily located on the anterior esophageal wall, anatomically adjacent to the LA and distant from the lower esophageal sphincter. Most lesions were solitary and appeared as linear ulcerations tracking along the ablation line or as “kissing” ulcers on opposing esophageal surfaces. As shown in Table S1, the morphology and incidence of EI differed by ablation modality. HPSD RFCA and CBA generally resulted in minimal or superficial lesions. CBA more frequently produced linear mucosal changes, while HPSD RFCA occasionally caused focal ulcerations. Across both techniques, the posterior LA wall remained the most vulnerable anatomical site.

### 3.5 Endoscopic Findings and Lesion Healing Course

**FIGURE 3:**
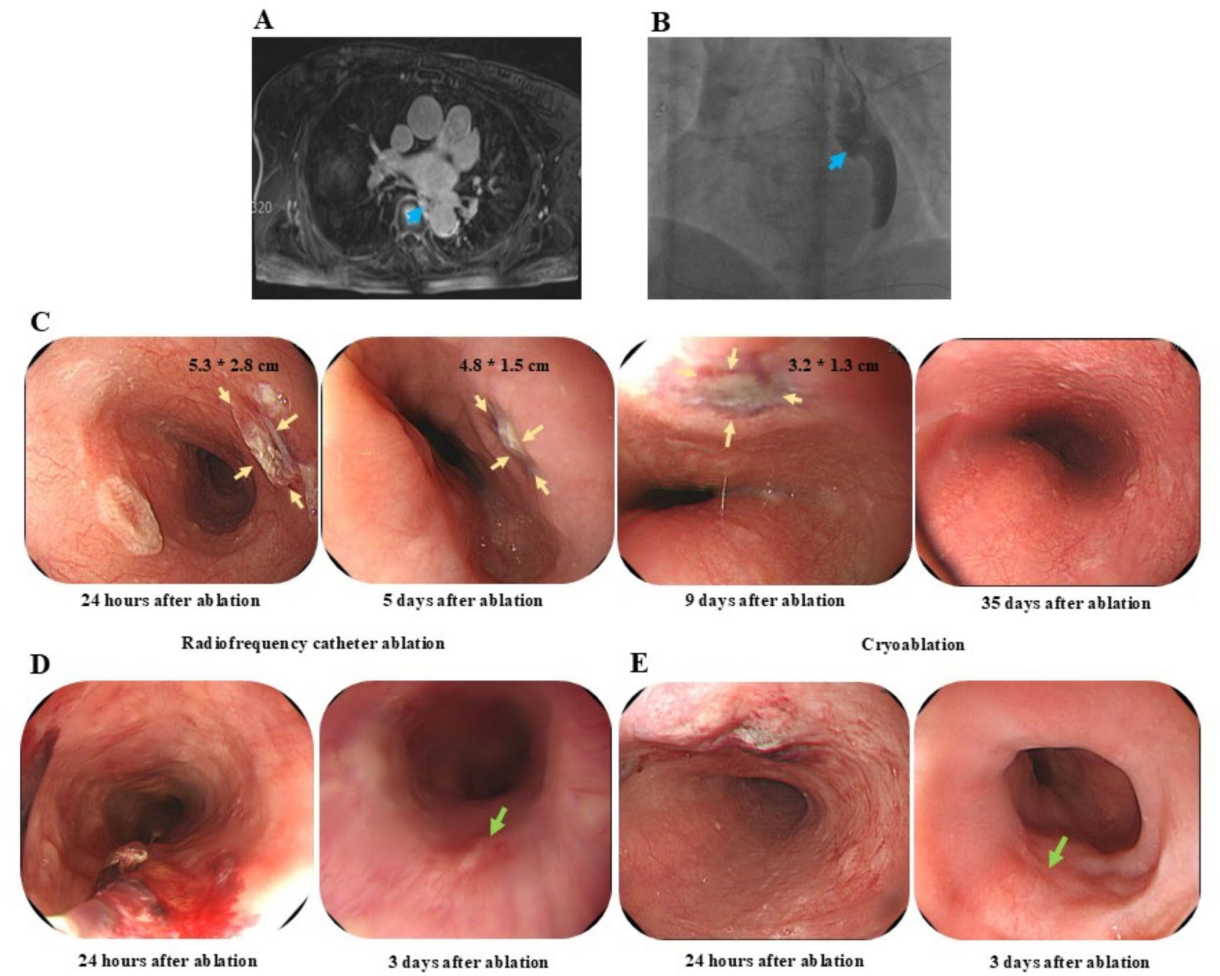
The healing process of esophageal injury. Healing of EI in patients treated with CA. (A) MRI showing compression of the esophagus by the LIPV. (B) Esophageal position confirmed by barium esophagography; most of the esophagus lies on the left side (blue arrow). (C) Endoscopic images post‐RFCA: On day 1, two superficial ulcerative lesions (largest: 5.3 × 2.8 cm) with surrounding congestion and edema were observed; high‐dose PPI therapy was initiated. By day 5, edema decreased. By day 9, lesions were smaller, and by day 35, complete healing was confirmed (yellow arrow: EI). (D, E) Comparison of lesions after RFCA and CBA. (D) RFCA‐induced superficial ulcer with hematoma. (E) CBA‐induced ulcerative lesion. EGD after 3 days of PPI therapy showed mucosal improvement (green arrow). No AEF developed. AEF, atrial‐esophageal fistula; CA, catheter ablation; CBA, cryoballoon ablation; EGD, esophagogastroduodenoscopy; EI, esophageal injury; LIPV, left inferior pulmonary vein; MRI, magnetic resonance imaging; PPI, proton pump inhibitor; RFCA, radiofrequency catheter ablation.

Figure 3 depicts the healing course of EI post‐CA. One day after RFCA, endoscopy revealed two superficial ulcerations (largest: 5.3 × 2.8 cm) with surrounding edema and hyperemia. PPI therapy has started. By day 5, edema had decreased; by day 9, the lesion was smaller, and complete healing was confirmed by day 35. MRI demonstrated esophageal compression by the LA (Figure 3A–C). Figure 3D,E compare lesions between RFCA and CBA. RFCA caused superficial ulceration with hematoma, while CBA showed discrete ulcers. Repeat EGD at 3 days post‐treatment showed marked mucosal improvement in both. Across the entire cohort, all 30 patients underwent follow‐up EGD at 7–10 days, with most demonstrating complete healing; only one CBA case had a small residual defect, and one RFCA case showed improving hematoma. Importantly, no patient developed AEF or required additional intervention during follow‐up. No AEF developed in any patient.

### 3.6 Risk Factors for Esophageal Injury Following Catheter Ablation

The results of logistic regression analysis assessing predictors of EI following CA are presented in Table 2. In the univariate analysis, a BMI of ≤ 24 kg/m^2^ (odds ratio [OR], 2.92; 95% confidence interval [CI], 1.39–6.12; *p* = 0.006) and an LVEF ≤ 40% (OR, 4.08; 95% CI, 1.56–10.69; *p* = 0.009) were significantly associated with the development of EI. Multivariate logistic regression confirmed these associations after adjustment that a lower BMI (OR, 3.20; 95% CI, 1.50–6.83; *p* = 0.003) and a reduced EF (OR, 4.74; 95% CI, 1.75–12.82; *p* = 0.002) were independent risk factors for EI following CA.

**TABLE 2:** Logistic regression analysis of esophageal injury after catheter ablation.

	Univariate		Multivariate	
	OR (95% CI)	*p*	OR (95% Cl)	*p*
Age > 60 (years)	1.29 (0.59–2.80)	0.523		
Female	1.10 (0.51–2.40)	0.792		
Height (cm)	1.03 (0.99–1.07)	0.216		
Body weight (kg)	0.99 (0.96–1.02)	0.410		
BMI ≤ 24 (kg/m^2^)	2.92 (1.39–6.12)	0.006	3.20 (1.50–6.83)	0.003
Persistent atrial fibrillation	1.52 (0.70–3.30)	0.290		
Hypertension	1.10 (0.52–2.32)	0.809		
Diabetes mellitus	0.97 (0.36–2.59)	0.946		
Stroke/TIA	1.55 (0.52–4.62)	0.428		
Vascular disease	2.57 (0.73–9.08)	0.131		
Congestive heart failure	1.15 (0.52–2.58)	0.726		
CHA_2_DS_2_–VASc ≥ 2	0.94 (0.44–2.02)	0.886		
LA size ≥ 40 (mm)	1.89 (0.76–4.72)	0.164		
LVEF ≤ 40 (%)	4.08 (1.56–10.69)	0.009	4.74 (1.75–12.82)	0.002
E/e'	0.94 (0.83–1.05)	0.271		
PAP (mmHg)	1.03 (0.98–1.08)	0.238		
LVEDD	1.06 (0.99–1.14)	0.100		
LA volume ≥ 100 (mL)	1.40 (0.63–3.12)	0.405		
TEE before RFCA	1.99 (0.89–4.49)	0.090		
CFAE	0.95 (0.93–0.97)	0.458		
Roof line	1.51 (0.34–6.70)	0.585		
PMI	0.80 (0.10–6.10)	0.826		
SVC	0.53 (0.12–2.27)	0.383		
Posterior wall ablation	1.00 (0.23–4.35)	0.998		
Lt PV additional ablation	1.90 (0.90–4.00)	0.087		

*Note:* Adjustment for age, female, BMI, TEE before RFCA, LVEF, LA volume, persistent atrial fibrillation, posterior wall ablation.

Abbreviations: BMI, body mass index; CFAE, complex fractionated atrial electrogram; LVEDD, left ventricular end‐diastolic diameter; LVEF, left ventricular ejection fraction; PV, pulmonary vein; RFCA, radiofrequency catheter ablation; SVC, superior vena cava; TEE, transesophageal echocardiography; TIA, transient ischemic attack.

## 4 Discussion

The main findings of this study are as follows: (1) EI occurred in 5.1% of patients undergoing CA, with comparable incidence between RFCA (5.0%) and CBA (6.3%). (2) Most lesions were minimal, such as erythema or superficial ulceration, and were effectively managed with high‐dose PPIs, without progression to AEF. (3) Lower BMI and reduced LVEF were independently associated with EI development. Early rhythm control using CA has been shown to improve symptom relief, sinus rhythm maintenance, and reduce cardiovascular outcomes compared with delayed intervention, supporting its increasing use [10]. However, CA is not without risk and may lead to serious complications such as stroke, cardiac tamponade, and AEF [11, 12]. Although rare, AEF is the most fatal complication with a high associated mortality rate [2], and even minor esophageal lesions, though often minimal in appearance, may progress to this life‐threatening condition. These findings highlight the need for early detection of EI to prevent its progression to life‐threatening outcomes.

### 4.1 Risk and Management of Esophageal Injury

While CA has become a cornerstone of early rhythm control for AF due to its efficacy in symptom relief, sinus rhythm maintenance, and cardiovascular risk reduction, the procedure is not without risks. AEF, though rare, remains the most feared complication due to its high mortality. Thus, early recognition and mitigation of EI are critical. Multiple strategies have been proposed to reduce EI risk, including preprocedural imaging (CT, MRI, barium esophagography) to determine esophageal position and real‐time esophageal temperature monitoring. However, esophageal movement during the procedure can be unpredictable. Good et al. reported that the esophagus can shift > 1 cm in 96% of patients and > 4 cm in 4% during ablation, complicating real‐time targeting [13]. Excessive contact force (> 20 g) may also increase the risk of steam pops without added benefit [14]. Moreover, esophageal temperature probes themselves may contribute to injury through conductive heating [15].

In addition to direct thermal mucosal injury, vagal nerve‐mediated gastric hypomotility has also been described as a potential complication of AF ablation [16]. Injury to the periesophageal vagal plexus may lead to transient gastric emptying disorders, particularly after posterior LA ablation. Collateral injury to this plexus, whether from RFCA or CBA, may impair gastric motility, leading to symptoms such as bloating or gastroparesis. Cryoablation induced gastroparesis has also been reported, particularly when the esophagus lies midline and close to the ablation zone [17]. Although our study focused on EI, this finding highlights the need for future studies to assess functional complications using objective tools such as gastric emptying scintigraphy. However, our study did not include a systematic assessment of symptoms or objective indicators of gastric or esophageal motility disorders (e.g., history of GERD, LES tone, or esophageal dilation), which we acknowledge as a limitation.

In this study, RFCA used lower power (25–30 W) compared to the HPSD protocol. While lower power may appear safer, it requires longer procedure times, which may increase the risk of conductive heat spread and EI. These biophysical differences should be considered when generalizing the results of this study to HPSD‐based centers. In our study, HPSD ablation was selectively used but not systematically recorded across all patients. Among those with esophageal injury, four had received HPSD, but the small number and incomplete documentation limited further subgroup analysis.

### 4.2 Distinct Mechanisms and Lesion Patterns of RFCA and CBA

The mechanisms of EI differ fundamentally between RFCA and CBA. RFCA induces thermal injury primarily through resistive heating at the catheter‐tissue interface, followed by conductive heat spread, which can cause transmural damage to adjacent structures. In contrast, CBA causes cellular destruction via intracellular ice crystal formation, microvascular compromise, and subsequent ischemic injury, while largely preserving the extracellular collagen matrix. These biophysical differences may contribute to distinct lesion patterns and healing processes between treatment modalities. Compared to RFCA, CBA preserves the extracellular collagen matrix despite its cytotoxic effect, which may explain the relatively favorable mucosal healing observed in CBA‐induced EI [18]. RFCA typically involves point‐by‐point energy delivery with higher contact force variability and greater energy titration control, whereas CBA delivers circumferential energy with less operator‐dependent variability. The anatomic relationship between the LA posterior wall and esophagus may be particularly relevant in RFCA, where deeper or more prolonged energy delivery could increase the risk of thermal injury. This biophysical difference may confer protective effects on esophageal integrity, though both modalities require careful energy application near the posterior LA. Given these distinctions, future studies and preventive strategies should consider ablation modality‐specific mechanisms when assessing EI risk, monitoring techniques, and procedural safeguards.

### 4.3 Role of BMI and LVEF in Predicting EI

Our study demonstrated that a BMI of ≤ 24 kg/m^2^ and a LVEF of ≤ 40% are significant independent risk factors for EI after CA. Anatomically, the fatty tissue between the esophagus and LA acts as a thermal insulator. Thinner fat layers, more likely in patients with low BMI, may allow greater transmission of ablative energy to the esophagus [1, 4]. Chahine et al. further demonstrated a direct association between BMI and epicardial adipose tissue (EAT) thickness overlying the LA [19]. Reduced LVEF is associated with LA remodeling, posterior wall thinning, and altered chamber geometry, potentially shortening the distance between the LA and esophagus and predisposing to injury. Although several patients in our cohort had cardiomyopathy, EI was primarily observed in those with systolic dysfunction rather than those with preserved EF, supporting the role of impaired contractility as a risk factor. Previous imaging studies have shown that a shorter LA‐esophagus distance independently predicts EI risk [20, 21]. Our findings reinforce the importance of patient‐specific anatomical and physiological factors when assessing EI risk pre‐procedurally.

### 4.4 Therapeutic Implications of PPI Use

While the pathophysiology of EI is multifactorial, including thermal injury, acid reflux, vagal dysfunction, and mucosal ischemia [22], our study supports the protective role of early PPI administration in limiting lesion progression. All EI patients were treated with high‐dose PPIs, which may have contributed to lesion healing and prevention of AEF progression. Schmidt et al. [23] reported that reflux‐like symptoms were related to changes in the esophageal wall.

### 4.5 Clinical Implications

AEF, although rare, is one of the most devastating complications following CA and typically presents 2–4 weeks post‐procedure. Given its high mortality, early identification of EI, a potential precursor to AEF, is essential. Our study demonstrates that patients with lower BMI and reduced LVEF are at elevated risk for developing EI. These patients may benefit from post‐ablation EGD for early lesion detection and timely intervention. To our knowledge, this is the first study to identify specific clinical predictors of EI following both radiofrequency and cryoballoon ablation in a real‐world cohort, providing valuable guidance for risk stratification and surveillance.

## 5 Limitation

This study has several limitations. First, it was a retrospective single‐center study, introducing potential selection bias and limiting causal inference. Second, most patients underwent RFCA, creating an imbalance that may reduce the power of CBA subgroup analysis. Third, the exclusively East Asian cohort may limit generalizability to populations with different BMI distributions. Fourth, BMI was used as a surrogate for periesophageal fat, as imaging data on esophagus‐to‐LA distance or fat thickness were unavailable. Other unmeasured factors, such as waist circumference, muscle mass, and fat distribution, may also influence EI risk. Lastly, procedural details, including contact force, lesion duration, and esophageal temperature, were not consistently recorded, limiting our ability to assess their influence on EI occurrence. Despite these limitations, this study provides novel insights into patient‐related risk factors for EI and underscores the value of targeted post‐ablation surveillance in high‐risk individuals.

## 6 Conclusion

Esophageal injury following catheter ablation for atrial fibrillation is not uncommon but generally mild in severity. A lower BMI and reduced LVEF were identified as independent risk factors for EI regardless of ablation modality. These findings suggest that patient‐specific anatomical and physiological characteristics contribute significantly to injury risk. Post‐procedural EGD may be warranted in high‐risk patients to facilitate early detection, guide therapeutic decisions, and prevent progression to serious complications such as AEF. This insight supports a more personalized approach to procedural planning and post‐ablation surveillance in AF management.

## Author Contributions

Yun Young Choi and Jong‐Il Choi developed the concept and design of the study. Yun Young Choi, Joo Hee Jeong, Chang Ok Seo, Yun Gi Kim, Jaemin Shim, Young‐Hoon Kim, and Jong‐Il Choi collected and analyzed the data. Yun Young Choi and Jong‐Il Choi wrote the initial draft of the manuscript. Hyuk Soon Choi commented on a draft of the manuscript. Statistical analyses were performed by Yun Young Choi. All authors have read and approved the final version of this manuscript.

## Funding

This work was supported by a Korea University Grant (J.‐I.C.), a grant from Korea University Anam Hospital, Seoul, Republic of Korea (J.‐I.C.), and in part, by grants from the Basic Science Research Program through the National Research Foundation of Korea funded by the Ministry of Science and ICT (2021R1A2C2011325 to J.‐I.C.). The funders had no role in data collection, analysis, interpretation, trial design, patient recruitment, or any aspect pertaining to the study.

## Conflicts of Interest

The authors declare no conflicts of interest.

## Supporting information


**Table S1:** Kansas City esophageal injury classification.
**Table S2:** Distribution of esophageal position in relation to the left atrium on computed tomography.
**Table S3:** Baseline characteristics of patients with esophageal injury between radiofrequency catheter ablation and cryoballon ablation.
**Table S4:** Cryablation procedural parameters by pulmonary vein in patients with and without esophageal injury.
**Figure S1:** Comparison esophageal injury between HPSD and cryoablation.

## Data Availability

The data underlying this article will be shared upon reasonable request from the corresponding author.
