# Supplementary material for: Esophageal Injury Risk Factors in Patients With Atrial Fibrillation Undergoing Catheter Ablation
Source: J Arrhythm. 2026 Feb 9;42(1):e70259. doi: 10.1002/joa3.70259 (PMC12884352; doi:10.1002/joa3.70259)
Supplement: Supplementary file 1 — Table S1: Kansas City esophageal injury classification. Table S2: Distribution of esophageal position in relation to the left atrium on computed tomography. Table S3: Baseline characteristics of patients with esophageal injury between radiofrequency catheter ablation and cryoballon ablation. Table S4: Cryablation procedural parameters by pulmonary vein in patients with and without esophageal injury. Figure S1: Comparison esophageal injury between HPSD and cryoablation. [file JOA3-42-e70259-s001.docx]

Supplementary

**eTable 1. Kansas City Esophageal injury Classification**

| **Kansas City Esophageal injury**  **Classification** | **Degree of injury** | **Confirm** |
| --- | --- | --- |
| Type 1 | Erythema and erosion | Endoscopy |
| Type 2a | Superficial ulcers involving mucosa alone ± clot | Endoscopy |
| Type 2b | Deep ulcer involving up to the muscularis externa ± clot | Endoscopy |
| Type 3a | Perforation without communication with atria | Identified on CT |
| Type 3b | Perforation with atrio-esopohageal fistula | Identified on CT |

CT, Computed tomography

**eTable 2. Distribution of Esophageal Position in Relation to the Left Atrium on Computed Tomography**

| **Ablation Type** | **Total patients** | **Esophagus on** | **Esophagus on** | **Esophageal** | **EI cases with** |
| --- | --- | --- | --- | --- | --- |
|  |  | **Left of LA, n (%)** | **Right/Midline, n (%)** | **injury cases** | **Left-sided esophagus, n (%)** |
| RFCA | 504 | 488 (96.8) | 16 (3.2) | 25 | 25 (100) |
| CBA | 80 | 79 (98.8) | 1 (1.2) | 5 | 5 (100) |
| Total | 584 | 567 (97.1) | 17 (2.9) | 30 | 30 (100) |

**eTable 3. Baseline characteristics of patients with esophageal injury between radiofrequency catheter ablation and cryoballon ablation**

| **Variables** | **RFCA** | **CBA** | **P-value** |
| --- | --- | --- | --- |
|  | **(n=25)** | **(n=5)** |  |
| Age, years | 63 ± 9.0 | 60.4 ± 8.2 | 0.554 |
| Male, n (%) | 15 (60) | 5 (100) | 0.001 |
| Body weight (kg) | 68.8 ± 15.3 | 75.8 ± 9.4 | 0.337 |
| Height (cm) | 167.9 ± 10.3 | 172.4 ± 3.7 | 0.109 |
| BMI, (kg/m2) | 24.3 ± 4.2 | 25.5 ± 2.4 | 0.555 |
| Paroxysmal atrial fibrillation, n (%) | 6 (24) | 4 (80) | 0.014 |
| Congestive Heart Failure, n (%) | 9 (36) | 0 (0) | 0.001 |
| Hypertension, n (%) | 14 (56) | 4 (80) | 0.324 |
| Diabetes, n (%) | 5 (20) | 0 (0) | 0.022 |
| Previous stroke, n (%) | 4 (16) | 0 (0) | 0.043 |
| Vascular disease, n (%) | 2 (80) | 1 (20) | 0.432 |
| CHA2DS2-VASc score | 2.4 ± 1.7 | 1.2 ± 0.8 | 0.140 |
| Left ventricular ejection fraction (%) | 47.6 ± 8.9 | 55.1 ± 4.1 | 0.081 |
| Left atrium diameter (mm) | 44.1 ± 4.8 | 35.8 ± 4.4 | 0.024 |
| E/e‘ | 8.7 ± 2.1 | 7.7 ± 1.6 | 0.329 |
| Pulmonary artery pressure (mmHg) | 32.5 ± 6.7 | 31.8 ± 3.6 | 0.740 |
| LVEDD (mm) | 48.5 ± 4.8 | 47 ± 3.5 | 0.506 |
| Left atrial volume (ml) | 115.8 ± 28.3 | 90 ± 15.8 | 0.060 |
| TEE before RFCA, n (%) | 7 (28) | 2 (40) | 0.608 |
| Ablation, n (%) |  |  |  |
| CTI | 20 (80) | 1 (20) | 0.006 |
| Lt PV additional ablation | 12 (48) | 1 (20) | 0.257 |

BMI, body mass index; AF, atrial fibrillation; LVEDD, left ventricular end-diastolic diameter; TEE, transesophageal echocardiography; RFCA, radiofrequency catheter ablation; CTI, cavotricuspid isthmus; PV, pulmonary vein.

**eTable 4. Cryablation procedural parameters by Pulmonary Vein in patients with and without Esophageal Injury**

|  | Total | EL (-) | EL (+) | P value |
| --- | --- | --- | --- | --- |
|  | n=80 | n=75 | n=5 |  |
| LSPV |  |  |  |  |
| Number of applications | 1.6±1.1 | 1.6±1.13 | 1±0.0 | <0.001 |
| Freeze Duration | 191.1±39.9 | 191.5±38.7 | 186±61.5 | 0.768 |
| Nadir Temperature | -48.6±6.4 | -48.9±6.4 | -44±5.1 | 0.097 |
| Time to isolation | 38.3±28.2 | 38.7±28.6 | 32.8±22.8 | 0.656 |
| LIPV |  |  |  |  |
| Number of applications | 1.5±0.7 | 1.5±0.8 | 1.2±04 | 0.337 |
| Freeze Duration | 204±35.1 | 204.8±34.3 | 192.0±50.2 | 0.435 |
| Nadir Temperature | -41.9±16.5 | -42.0±17.1 | -42.4±2.3 | 0.953 |
| Time to isolation | 35.8±32.8 | 36.3±33.3 | 28.6±25.2 | 0.615 |
| RSPV |  |  |  |  |
| Number of applications | 1.9±1.3 | 1.9±1.3 | 1.2±0.5 | 0.231 |
| Freeze Duration | 168.7±36.8 | 167.9±37.9 | 180±0.0 | 0.007 |
| Nadir Temperature | -50.2±9.6 | -50.4±9.8 | -47.2±3.9 | 0.473 |
| Time to isolation | 19.2±19.0 | 19.3±19.4 | 19±13.1 | 0.977 |
| RIPV |  |  |  |  |
| Number of applications | 2.1±1.4 | 2.1±1.4 | 3.0±0.7 | 0.136 |
| Freeze Duration | 182.7±55.1 | 182.0±56.5 | 192.0±26.8 | 0.698 |
| Nadir Temperature | -42.9±17.6 | -43.0±18.1 | -40.8±6.4 | 0.787 |
| Time to isolation | 29.3±38.9 | 28.8±38.4 | 36.4±49.9 | 0.674 |

LSPV, left superior pulmonary vein; LIPV, left inferior pulmonary vein; RSPV, right superior pulmonary vein; RIPV, right inferior pulmonary vein; EL, esophageal lesion

**
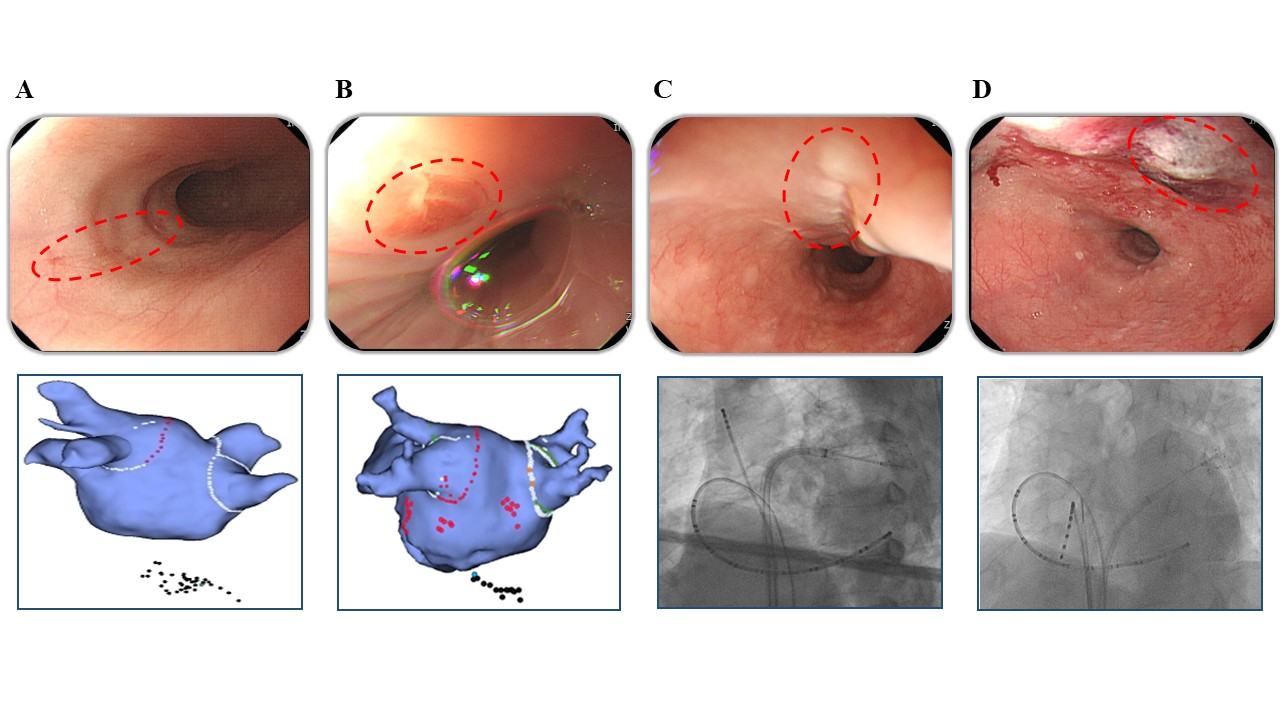
eFigure 1. Comparison esophageal injury between HPSD and cryoablation**

**A-B.** CA with HPSD resulted in fewer lesions and less severe lesions than non-HPSD ablation: **A**. RFCA targeting the left posterior pulmonary vein with HPSD. **B.** HPSD RFCA with additional low-voltage-guided posterior wall ablation. **C-D**. Representative lesions following CBA: **C**. Minimal linear lesions. **D**. Superficial ulcerative lesions. Red dotted circles indicate the location of EIs observed after HPSD and CBA.

EI, esophageal injury; CA, catheter ablation; HPSD, high-power short-duration; RFCA, radiofrequency catheter ablation; CBA, cryoballoon ablation.
